# Current Advances in Mitochondrial Targeted Interventions in Alzheimer’s Disease

**DOI:** 10.3390/biomedicines11092331

**Published:** 2023-08-22

**Authors:** Tiago Sousa, Paula I. Moreira, Susana Cardoso

**Affiliations:** 1 Faculty of Medicine, University of Coimbra, 3000-370 Coimbra, Portugal; sousarodriguestiago@gmail.com; 2 CNC—Center for Neuroscience and Cell Biology, University of Coimbra, 3004-504 Coimbra, Portugal; pimoreira@fmed.uc.pt; 3 CIBB—Center for Innovative Biomedicine and Biotechnology, University of Coimbra, 3004-504 Coimbra, Portugal; 4 Institute of Physiology, Faculty of Medicine, University of Coimbra, 3000-370 Coimbra, Portugal; 5 IIIUC—Institute for Interdisciplinary Research, University of Coimbra, 3030-789 Coimbra, Portugal

**Keywords:** Alzheimer’s disease, glucose metabolism dysregulation, mitochondria, mitochondrial-directed approaches, oxidative stress

## Abstract

Alzheimer’s disease is the most prevalent neurodegenerative disorder and affects the lives not only of those who are diagnosed but also of their caregivers. Despite the enormous social, economic and political burden, AD remains a disease without an effective treatment and with several failed attempts to modify the disease course. The fact that AD clinical diagnosis is most often performed at a stage at which the underlying pathological events are in an advanced and conceivably irremediable state strongly hampers treatment attempts. This raises the awareness of the need to identify and characterize the early brain changes in AD, in order to identify possible novel therapeutic targets to circumvent AD’s cascade of events. One of the most auspicious targets is mitochondria, powerful organelles found in nearly all cells of the body. A vast body of literature has shown that mitochondria from AD patients and model organisms of the disease differ from their non-AD counterparts. In view of this evidence, preserving and/or restoring mitochondria’s health and function can represent the primary means to achieve advances to tackle AD. In this review, we will briefly assess and summarize the previous and latest evidence of mitochondria dysfunction in AD. A particular focus will be given to the recent updates and advances in the strategy options aimed to target faulty mitochondria in AD.

## 1. Introduction

Alzheimer’s disease (AD) is known to be the most common form of dementia among the elderly population, representing an estimated 60% to 80% of diagnosed dementia cases [1]. Although a small percentage of AD cases (approximately 1–5%) can be attributed to a genetically inherited predisposition and be diagnosed between 30 and 60 years old, the vast majority of AD cases are sporadic, being frequently diagnosed at over 65 years old [2]. The exact cause of the sporadic form of AD is not fully understood, but it is believed to have a multifactorial component involving both genetic and environmental factors that combine and move along a continuum until disease diagnosis [3]. In addition, it is also recognized that AD has a long asymptomatic pre-clinical phase, during which pathophysiological changes occur without noticeable cognitive symptoms. As the disease evolves, individuals start to perceive a gradual memory loss, cognitive decline, and behavioral changes that ultimately impair their daily routines [4]. Canonically, the pathological diagnosis of AD relies on the postmortem detection of the accumulation of abnormal structures, such as senile plaques resulting from the extracellular deposition of amyloid β protein (Aβ) and the intracellular accumulation of neurofibrillary tangles composed of hyperphosphorylated tau (pTau) protein, which could trigger the neurodegenerative cascade leading to the loss of neurons associated with higher brain function [4].

Despite the alarming numbers and the latest estimations suggesting that, by the year 2050, there will be more than 100 million people diagnosed with this disorder, AD continues to be a disease without an effective treatment. However, there are several ongoing attempts to modify the disease course, including the recently U.S. Food and Drug Administration (FDA)-approved drugs ducanumab and lecanema, both targeted to Aβ [5].

Considering the enormity of the problem, several countries have implemented a deadline to develop a cure or approved disease-modifying therapy to tackle AD [6,7]. In this scenario, recent reports highlight that the cognitive performance of individuals at older ages does not necessarily correlate with the accumulation of AD hallmarks [8], which highlights the need to move away from targeting the cardinal AD neuropathological hallmarks to identify and characterize the early brain changes in AD, in order to identify possible novel therapeutic targets to circumvent AD’s cascade of events. One of the most auspicious targets is mitochondria, conserved organelles indispensable in nearly all cells of the body [9]. Mitochondria are known as the cell’s powerhouses, responsible for generating the majority of the cell’s energy in the form of adenosine triphosphate (ATP) through oxidative phosphorylation. Beyond this role, mitochondria exert multiple essential functions in different cellular processes, e.g., the regulation of calcium homeostasis, reactive oxygen species formation (ROS), cell repair and cell death processes, intracellular signaling, and cell metabolism, among others [10]. In the brain, mitochondria acquire an even more important role, as the brain’s energy requirements, particularly of neuronal cells, are extremely high in order to support all of the complex processes and mechanisms necessary to ensure its adequate activity [11]. A vast body of literature has shown the occurrence of extensive mitochondria abnormalities in the continuum of AD-related brain events [12,13]. For instance, pivotal studies have revealed that during the transitional clinical stage, known as mild cognitive impairment (MCI), patients present a faulty cerebral energy metabolism, disturbances in the expression and/or activities of important regulatory mitochondrial energy-related enzymes (e.g., pyruvate dehydrogenase (PDH), isocitrate dehydrogenase, and α-ketoglutarate dehydrogenase (α-KGDH), and cytochrome oxidase (COX)), a significant rise in oxidative stress markers, and alterations in mitochondria number, shape, and localization [14,15]. Recently, mitochondria deficits were identified as a key trigger for the loss of synaptic function observed in cortical tissue from post-mortem AD patients [16]. In further agreement, data from model organisms of the disease reinforce the notion that mitochondria dysfunction and metabolic alterations precede AD neuropathology [17,18,19]. According to these premises, preserving mitochondria’s health and function could represent the “holy grail” to address AD’s prevalence. In this review, we will assess and summarize the previous and latest evidence of mitochondria disturbances in AD. A particular emphasis will be placed on the recent advances and updates in the treatment options aimed to target faulty mitochondria in AD. To perform this narrative review, the search was conducted during March–June 2023 in the database PubMed. Relevant articles were searched using different combinations of the keywords “Alzheimer’s disease” (AND) “mitochondria” (AND) and (OR) “mitochondria-directed strategies” (OR) “mitochondria” (AND) “brain health” (AND) “energy metabolism”. We also used the database ClinicalTrials.gov to search for research that has advanced into clinical trials. Herein, the keywords used were “Alzheimer’s disease” (AND) “mitochondria”. The search performed was narrowed to articles written in English and was independently undertaken by two authors (Tiago Sousa and Susana Cardoso). The article selection was performed without year limitations but, since we aimed to provide an updated overview of the current literature available on the subject, preference was given to articles published during the last ten years.

## 2. Mitochondria (Dys)Function in Alzheimer’s Disease: A Brief Overview of the Main Mechanisms

**Figure 1:**
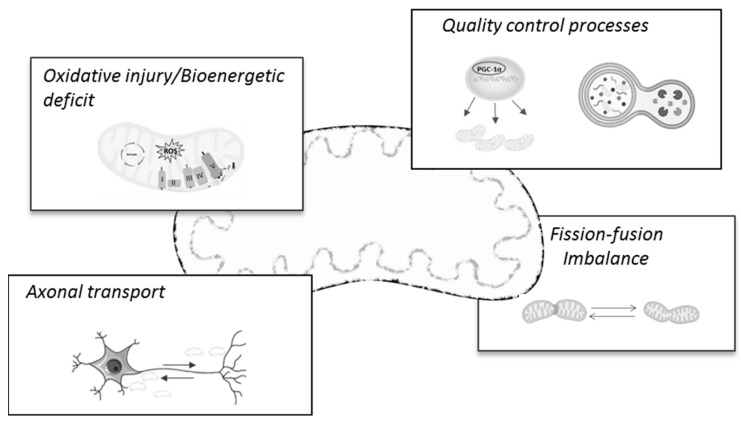
A schematic illustration of the main mitochondrial-related processes disturbed in Alzheimer´s disease. As is widely documented, Alzheimer’s disease (AD) (brain) is characterized by the loss of mitochondria homeostasis. This is mainly manifested by disturbances in the interconnected mechanisms of mitochondrial quality control (autophagy and mitochondrial biogenesis), mitochondrial axonal transport, fission/fusion processes and bioenergetics (energy production), and reactive oxygen species production (ROS). I—Complex I of the mitochondrial respiratory chain; II—Complex II of the mitochondrial respiratory chain; III—Complex III of the mitochondrial respiratory chain; IV—Complex IV of the mitochondrial respiratory chain; V—Complex V of the mitochondrial respiratory chain.

The first insights into mitochondria involvement in AD pathology emerged several years ago, when the electron microscopy analysis of the frontal cortex tissue of AD patients revealed the presence of abnormal mitochondria in apparent normal dendrites, but which were supposed to degenerate in later stages of the disease [20,21]. Since then, abundant research has demonstrated mitochondria and mitochondria-related mechanisms and processes as prominent features of the AD pathology and/or progression (for a review, see [22]). Notably, there is mounting evidence suggesting that many of these brain mitochondrial changes also occur in non-brain samples, such as peripheral blood lymphocytes [23], skin fibroblasts [24], peripheral blood mononuclear cells (PBMCs), and platelets [25]. In fact, a very recent work by Mahapatra and colleagues [25] confirmed that peripheral blood cells’ bioenergetics profiles closely correlated with subjects’ cognitive status across AD progression. In line with this, others report that the peripheral alterations of oxidative stress markers could represent feasible biomarkers for early AD diagnostics [26,27,28,29], thus highlighting and reinforcing the mitocentric view of AD. In the next subsections, we will provide a brief and up-to-date review of the main mitochondrial mechanisms found to be deregulated and/or impaired during the AD continuum, which will then be considered as possible targets for AD therapy (Figure 1).

### 2.1. Relevance of Oxidative Stress and Energy (Hypo)Metabolism in AD

Mitochondria are double-membrane-bound organelles with an outer membrane (OMM) facing the cytosol and a large folded inner mitochondrial membrane (IMM) with high selectivity capacity, which makes it impermeable to almost all molecules found in the matrix [10]. The oxidative phosphorylation system (OXPHOS) engrained in this membrane has the capacity to empower oxygen consumption with coupled ATP production through the coordination of five enzymatic complexes. Briefly, during cellular respiration, electrons flow through the mitochondrial electron transport chain (ETC) due to many redox reactions, forming an electrochemical gradient, which leads to ATP synthesis in complex V. If an electron escapes from the ETC, mainly in complexes I and III, it will subsequently bind oxygen (O_2_) and form superoxide (O_2_^−^), an anionic free radical (for a review, see [30]). Alongside O_2_^−^, hydroxyl radicals (^·^OH) and hydrogen peroxide (H_2_O_2_) constitute the reactive oxygen species (ROS) and are considered the main by-products of oxidative phosphorylation [31]. ROS are free radical and non-radical molecules that have the capacity to rapidly affect cell functionality and viability by damaging proteins, lipids, and nucleic acids, including mitochondrial structures that, due to the close production site, are not only the main producers but also the first targets of ROS-induced damage [31]. Importantly, it is also recognized that low/moderate concentrations of ROS are necessary for the regulation of different processes, such as cellular signaling, immune responses, and inflammation [32]. Thus, it is important to guarantee that ROS production and elimination exist in a highly controlled equilibrium (for a review, see [33]). To accomplish this, cells are equipped with a very efficient antioxidant system composed of enzymatic (e.g., superoxide dismutase (SOD), glutathione peroxidase (GPx), catalase) and non-enzymatic defenses (e.g., glutathione, vitamins C and E) [31]. This becomes of particular importance regarding brain homeostasis, which, due to its inherent properties, e.g., high content of polyunsaturated fatty acids (PUFA), high oxygen consumption, and modest antioxidant defense levels, is an organ that is extremely vulnerable to the deleterious effects of oxidative injury [34]. As with AD, plentiful evidence has confirmed that oxidative stress has a pivotal role in the pathogenesis of the disease [35]. As previously reported, relative to non-diseased brains, AD brain tissue, especially brain regions with histopathological features of the disease, possesses high levels of oxidative stress markers [36]. In particular, protein oxidation markers (e.g., protein carbonyls and 3-nitrotyrosine) [37] and lipid peroxidation products (e.g., malondialdehyde (MDA) and 4-hydroxynonenal (HNE)) have been found to be elevated in the vulnerable regions of AD brains [38,39], whilst, in contrast, earlier studies show significant alterations in the antioxidant defense system in AD brains [40]. Further in vivo studies corroborate the relevant role of ROS in AD [41] and report a negative correlation between cerebral ROS levels and mitochondrial function and learning and memory in mAPP mice at an early age [41]. In fact, it has been observed that oxidative deregulation, particularly oxidative-associated damage on mitochondria, can represent a pivotal event in the transition from a normal state to cognitive impairment [42], which can precede other pathological features of AD [43,44]. This view is further supported by the work of Leuner and colleagues [45], which, by means of in vitro and in vivo models of AD, showed that mitochondria-derived ROS are able to trigger the amyloidogenic amyloid precursor protein (APP) processing, leading to Aβ formation, thus demonstrating the strong influence that mitochondria and Aβ can have on each other [46]. In this context, a previous study revealed a close relation between the degree of cognitive dysfunction observed in transgenic AD mice and the range of synaptic mitochondrial dysfunction and mitochondrial Aβ levels [47]. Consistent with these observations, earlier data indicated that extracellular Aβ can be internalized by cells and imported into mitochondria cristae via the translocase of the outer membrane (TOM) machinery [48]. Inside mitochondria, similar works demonstrate that Aβ interacts with mitochondrial 17β-hydroxysteroid dehydrogenase type 10 (HSD10), an enzyme present in neuronal mitochondria [17] and often referred to as Aβ-binding alcohol dehydrogenase (ABAD), and also with Presequence protease (PreP), a mitochondrial Aβ-degrading enzyme localized in the mitochondrial matrix [49]. However, whilst the interaction of Aβ with HSD10 causes a prompt increase in ROS levels, namely O_2_·^−^ and H_2_O_2_, and a loss of mitochondria function [50], PreP activity was found to be decreased in AD brains and AD transgenic mouse brains, which also presented higher levels of oxidative products in their mitochondria [49]. It has been therefore proposed that modulating PreP activity could be a feasible strategy to control the mitochondrial Aβ content and mitochondrial oxidative stress [51,52]. Moreover, a recent in vitro study performed in iPSC-derived neurons and SY5Y cells transfected with an AβPP construct identified an undescribed correlation between the mitochondrial membrane potential, AβPP mitochondrial localization, and Aβ secretion [53]. In detail, the authors observed that depolarized mitochondria had more AβPP, secreted less Aβ, and consequently had higher intracellular Aβ levels; the opposite occurred when mitochondria were hyperpolarized (i.e., membrane potential was high), suggesting that Aβ secretion can represent a feasible biomarker of cell or tissue mitochondrial health [53]. In order to further explore the effects of the mitochondria–AD hallmark interaction, Rhein and colleagues [54] conducted a quantitative proteomic analysis and functional assays in four different strains of mice: the single transgenic pR5 mice to model tau pathology, the double transgenic APP/PS2 that model the Aβ plaque pathology, and the triple transgenic mice (pR5/APP/PS2) that combine the Aβ and tau pathologies. Among the main findings of their study, the authors observed a distinct pattern in Aβ and tau’s effects on OXPHOS components; for the same age, complex I activity was preferably affected in the tau mice model, whilst complex IV activity was firstly compromised in the APP/PS2 mice [54]. With advancing age, the triple transgenic strain presented the significant aggravation of the mitochondrial respiratory capacity, impaired mitochondrial enzyme activity, and lower ATP levels—alterations that were further associated with pronounced oxidative stress and, in line with other studies [55], disclosed the synergistic effects of both Aβ and tau on mitochondria. Following the same line of investigation, a recent proteomics study on AD post-mortem tissue observed a differentially altered pattern of mitochondrial electron transport chain components in age-dependent AD [56]. Specifically, the data obtained revealed the significant destabilization of complex I and the downregulation of subunits of complexes II, III, and V in the mitochondria of early-onset AD individuals, whilst complex IV subunits were only downregulated in AD aged mitochondria [56].

Previous research has shown that oxidative damage is closely interrelated with impairments in brain energy metabolism, particularly glucose metabolism [57,58]. Indeed, the use of proteomics analysis in MCI, in early and late AD brain tissue, made it possible to detect oxidative modifications in proteins involved in the glycolytic pathway [37,59], in the tricarboxylic acid (TCA) cycle [60], and in components of the respiratory chain [61], which, by causing proteins´ dysfunction, are believed to contribute to cellular energetic compromise and glucose hypometabolism [62]. In addition, evidence gathered from an in vitro study in which hippocampal and cortical primary neurons were exposed to increasing concentrations of Aβ_25–35_ suggested a strong link between the impairment of glucose transport and the neurotoxicity of the peptide [58]. In detail, the authors reported that the observed Aβ-mediated inhibition of glucose transport was causally related to Aβ-induced membrane lipid peroxidation and the generation of HNE [58]. Following these premises, and in agreement with prior neuroimaging studies using positron emission tomography (PET) imaging with 2-[^18^F] fluoro-2-deoxy-d-glucose ([^18^F]FDG) [63,64,65,66], a recent study has shown that brain glucose deregulation is undeniably an important early event in AD pathogenesis [67]. Using the well-characterized cohort of the Baltimore Longitudinal Study of Aging as an experimental group, An and colleagues [67] were able to observe a significant correlation between the brain tissue glucose concentration and the severity of AD hallmarks—an outcome that was associated with lower activity of the principal rate-controlling enzymes of glycolysis (hexokinase, phosphofructokinase, and pyruvate kinase) and lower expression levels of the neuronal glucose transporter 3 (GLUT3). In a complementary approach, Terada and colleagues, by means of updated neuroimaging techniques, disclosed the distinct contributions of glycolysis and mitochondrial OXPHOS activity to AD brain hypometabolism in the living brains of AD individuals [68]. The data obtained revealed an early decrease in mitochondrial oxidative activity in patients’ parahippocampi, detected using a specific PET probe for mitochondrial complex I, which was not accompanied by alterations in overall glucose metabolism, measured with the [^18^F]FDG tracer, thus suggesting that mitochondria-related energy failure may precede glycolysis-related hypometabolism in AD brains [68]. Consistent with this evidence, results obtained in the 5xFAD mouse model indicated that disturbances in mitochondria TCA cycle activity and mitochondria morphology in the hippocampi of young 5xFAD mice significantly impacted synaptic integrity during early amyloid pathology in this AD model [69]. Importantly, the authors’ experiments also disclosed an intricate interplay between hippocampal neurons and astrocytes’ metabolic function; herein, hippocampal astrocytes showed hampered glucose metabolism, which affected the glutamine supply to neuronal cells, impairing neuronal GABA synthesis and possibly the balance of synaptic excitation and inhibition in the AD brain, as previously reported [70]. 

Previous reports corroborate that, despite having a distinct metabolic profile (glycolytic for astrocytes and oxidative for neurons) characterized by cell-specific enzymatic features [71,72] and the organization of the mitochondrial respiratory chain [73], astrocyte–neuron coordination is of the utmost importance for the maintenance of brain function (for a review, see [74]). As an example, Vicente-Gutierrez and colleagues [75,76] demonstrated that astrocytic mitochondrial ROS production critically contributed to supporting brain energy metabolism, redox balance, neuronal function, and mice’s cognitive behavior. Concurrently, it has also been verified that an elevation in astrocytic aerobic glycolysis improves astrocytes’ support of neurons, thereby promoting neuronal survival and axon growth [77]. Aerobic glycolysis, i.e., the non-oxidative metabolism of glucose despite the presence of oxygen, has been considered a critical mechanism for neuroprotection, synaptic function, and rapid energy generation for cognitive task performance, etc., being associated with astrocytes’ glycolytic profiles [78] and often found to be decreased during the aging process [79]. In this line of investigation, recent findings identified an important association between the preservation of aerobic glycolysis and initial resilience to amyloid pathology, whilst, in contrast, the loss of this pattern correlated with cognitive impairment [80]. In this context, previous in vitro observations reveal that impaired astrocytic glycolysis results in the increased accumulation of Aβ within and around astrocytes and in the greater vulnerability of these cells to Aβ toxicity. In a similar manner, the impairment of glucose metabolism, mainly due to lower aerobic glycolysis, was linked with higher tau protein deposition in the vulnerable brain regions of pre-clinical and mildly symptomatic AD patients [81]. Overall, this evidence highlights the important impact that deregulated brain glucose metabolism can exert in the context of AD, suggesting new potential directions for AD treatment.

### 2.2. Mitochondrial Dynamics and Transport Alterations in AD

Mitochondria are described to be dynamic organelles with a striking ability to adapt their morphology, size, and location within the cell in order to maintain cellular requirements and proper function [82]. To successfully accomplish this task, the mitochondrial network relies on the balance between two opposing processes, fusion and fission, both regulated by several proteins belonging to the family of GTPases [83]. In a concise manner, under physiological conditions, mitochondrial fusion, regulated by mitofusins 1 and 2 (Mfn1 and Mfn2) located in the outer mitochondrial membrane and by optic dominant atrophy 1 (OPA1) present in the inner mitochondrial membrane, allows the cellular content and mitochondrial DNA to be combined to form a more resource-laden subcellular compartment. Unopposed fission, driven by the activity of the dynamin-related GTPase protein (Drp1) and mitochondrial fission 1 (Fis1), is essential to promote the conversion of damaged mitochondria into fragments, enabling their transport and selective degradation by mitophagy [84]. Altered mitochondria dynamics towards increased mitochondrial fission and consequent mitochondria fragmentation have been well established in AD [85]. This increased mitochondrial fragmentation was found to evolve as the disease progresses and was suggested to be related to the colocalization of Drp1 with Aβ monomers and oligomers in the postmortem brain tissue of AD patients [86]. Similarly, others reported increased mitochondrial fission in response to the increased production of Aβ in M17 cells and rat primary hippocampal neurons overexpressing APP [87]. Moreover, post-translational modifications of Drp1, such as increased S-nitrosylation [88] and phosphorylation at specific residues (e.g., ser616 and ser579) [89,90,91], were found to contribute to mitochondria dysfunction and AD pathology. More recently, a new mitochondrial phenotype characterized by elongated interconnected organelles was observed in the brain tissue of AD patients, animal models of AD, and wild-type aged mice [92]. This previously unknown morphology was designated as mitochondria-on-a-string (MOAS) and is believed to occur due to fission arrest at the final stages of the fission process as an attempt to preserve the remaining mitochondrial function in response to energetic stress [92]. Such a mitochondrial phenotype was later observed in the brains of OXYS rats, a model of sporadic AD [93], and in aged non-human primates [94]. Very recently, Panes and colleagues [95] reported that the MOAS morphology represented approximately 80% of the total mitochondria in the hippocampi of APP/PS1 mice. Moreover, they observed a significant interaction between MOAS and endoplasmic reticulum (ER) membranes, forming extensive mitochondria–ER contact sites (MERCS). Although the exact role of such an interaction remains unclear, the authors showed that reestablishing energy homeostasis reduced MOAS formation, MERCS coverage, and ER stress and enhanced the mitochondrial dynamics [95]. Alongside alterations in the CNS, previous studies refer to the existence of an abnormal mitochondrial distribution and morphology in fibroblasts from sporadic AD cases due to a reduction in Drp1 protein levels [96]. Additionally, others report slower mitochondria dynamics in sporadic AD fibroblasts due to the downregulation of both fission- and fusion-related proteins [97]. In further support, Drabik and colleagues [98] found that, in comparison with control patients, fibroblasts from sporadic AD individuals had lower levels of the fusion-regulating proteins and of Drp1, which were accompanied by a lower rate of mitochondrial fusion–fission. These alterations evoked the appearance of a less branched mitochondrial network characterized by less separated mitochondria that presented a smaller size. Although these findings suggest a less fragmented phenotype and oppose the alterations detected in brain tissue or in vitro models, they support the notion that mitochondrial dynamics have a prominent role in AD pathogenesis [99].

Intrinsically related to mitochondrial dynamics is the transport of mitochondria within neurons [100]. This motility is essential to provide energy in the form of ATP to neuronal synapses (anterograde transport driven by kinesin-1 (KIF5) motors) and to ensure the transport of aged or dysfunctional mitochondria back to the cell body, enabling its degradation (retrograde transport mediated by dynein motors) [101]. In AD, both in vivo and in vitro models demonstrate that the impairment of mitochondrial neuronal transport and the consequent energy deficit is one of the earliest events leading to the loss of axonal integrity and synaptic function [16,102,103,104]. For instance, it was reported that the lack of presynaptic mitochondria may underlie the selective regional loss of cortical synapses in the 5xFAD mouse model [105] and in AD brain tissue [106]. From a mechanistic point of view, earlier studies show that the combined overexpression of truncated tau with Aβ treatment in primary neurons promotes an impairment in mitochondrial transport by increasing the stationary mitochondrial population [107]. In further support, Vossel and collaborators found that the tau protein mediates Aβ-induced mitochondrial axonal transport impairment [108]. Using different experimental settings, the authors were able to verify that Aβ’s effects on mitochondrial transport involve glycogen synthase kinase 3beta (GSK3β) signaling [108], a kinase with proven involvement in Aβ-induced anterograde axonal transport deficits through its interaction with kinesin light chains [109]. More recently, it was demonstrated that the overexpression of tau P301L in primary neurons inhibited kinesin recruitment to mitochondria, thus inhibiting the anterograde mitochondria’s movement towards the axon terminals [110]. Notably, the retrograde transport of mitochondria was not affected by P301L [110]. In fact, several data suggest the increased vulnerability of the anterograde axonal mitochondrial transport in comparison with the opposite retrograde movement in AD-related conditions [103]. It was found that the expression content of KIF5A, a neuronal kinesin-1 isoform, and Miro1, an adaptor protein required for the axonal transport of mitochondria, was significantly decreased in the temporal lobes from postmortem AD brains, whilst dynein expression remained unchanged [111]. Similar findings were further observed in the neocortex in 5xFAD mice and in Aβ-treated primary neurons, whilst, in contrast, KIF5A overexpression in Aβ-treated neurons restored mitochondrial motility and attenuated neuronal oxidative stress and the loss of synaptic markers [111]. Consistently, others demonstrated that the knockdown of Miro1 or Milton, another adaptor protein, promoted the loss of axonal mitochondria in transgenic *Drosophila* expressing human tau [112]. As a result, the loss of mitochondria in axons was associated with the increased phosphorylation of the tau protein at AD-related residues, culminating in enhanced tau toxicity [112]. Following this line of investigation, Miro overexpression in AD transgenic flies was found to modulate mitochondria dynamics towards increased fusion, which correlated with increased ATP levels and decreased ROS levels [113], which supports the interlinked nature of mitochondrial quality control mechanisms.

### 2.3. Mitochondrial Biogenesis and Mitophagy

In concert with mitochondrial trafficking and dynamics, mitochondrial quality control relies on the balance between two complementary processes, the biogenesis of healthy and functional mitochondria and mitophagy, a selective mechanism where damaged or dysfunctional mitochondria are removed via autophagy [114]. In simple terms, mitochondria biogenesis is considered a self-renewal process that requires the participation of both mitochondrial and nuclear genomes, which interact in an organized multistep process [115]. The key components of mitochondrial biogenesis comprises peroxisome proliferator activated receptor-gamma (PPARα coactivator-1alpha (PGC-1α); two key nuclear transcription factors, nuclear respiratory factor 1 and 2 (NRF1 and NRF2); and the mitochondrial transcription factor A (Tfam). The sequential activation of each factor allows the integrative regulation of the nuclear-to-mitochondria proteins, thereby allowing the subunits of the respiratory chain to be encoded and contributing to the regulation of the antioxidant profile and mtDNA replication and transcription [116,117,118,119]. Previous and current studies demonstrate that compromised mitochondria biogenesis greatly contributes to mitochondrial impairment in AD [120,121]. Using postmortem brain samples from AD individuals and transgenic cells overexpressing APP mutations, Sheng and colleagues observed that the protein content of the critical regulators of mitochondrial biogenesis, PGC-1α, NRF1, NRF2, and TFAM, was significantly decreased in the AD milieu [120]. Additionally, the authors verified that the manipulation of PGC-1α expression in mutant M17 cells caused overt alterations in mitochondria function; if overexpressed, PGC-1α restored not only mitochondria biogenesis but also mitochondria ATP levels and complex IV activity, and opposite effects were noticed with PGC-1α silencing [120]. Concomitantly, data from human hippocampal brain samples of AD patients indicate that PGC-1α mRNA expression levels decrease as a function of the progression of clinical dementia [122]. Likewise, the PGC-1α protein content was inversely correlated with the Aβ peptide content and density of neuritic plaques [122]. In agreement with this, an in vivo study performed in 3xTg-AD mice at different ages revealed that the protein content and mRNA expression levels of mitochondrial biogenesis markers were already significantly decreased at 1 month of age, when mice did not present AD pathological hallmarks. Moreover, data obtained suggested that such earlier alterations were related to reduced mitochondria function at later ages, which prompted the authors to propose that disturbances in this quality control process precede the development and manifestation of AD pathology [121]. In further agreement, another study performed on the APP/PS1 mice model showed earlier changes affecting mitochondria biogenesis in the hippocampi of transgenic mice, prior to the formation of senile plaques and memory loss [123], thus reinforcing the idea that mitochondria malfunction, particularly in mitochondrial biogenesis, is an earlier event in the AD cascade. Accordingly, experimental evidence obtained from in vitro studies shows that Aβ_1–42_ peptide addition to neural stem cell cultures significantly compromises diverse mitochondrial parameters (e.g., ATP levels, mitochondrial ROS scavenging system, and mitochondrial dynamics), reduces mtDNA copy numbers, and downregulates the protein content of mitochondrial biogenesis regulators (PGC-1α, NRF1, and TFAM) [124]. In agreement with earlier works [125], the authors were able to observe that, when in culture, Aβ_1–42_ colocalizes with the mitochondrial network, suggesting a direct effect of Aβ on mitochondrial function. In addition, Dong and colleagues observed that hippocampal neurons exposed to Aβ_25–35_ experienced a significant decrease in the protein content of key mediators of mitochondria biogenesis and mtDNA levels, alongside the inhibition of the AMP-activated protein kinase (AMPK)–sirtuin 1 (SIRT1)–PGC-1α pathway [126]. Although the authors did not provide direct evidence of a causal link between AMPK–SIRT1–PGC-1α inhibition and Aβ_25–35_-induced mitochondrial biogenesis in hippocampal neurons, their findings support previous evidence about the important role that this signaling pathway has in rescuing mitochondrial defects by activating mitochondrial biogenesis in AD [126,127]. 

Alongside mitochondrial biogenesis, the other pillar of the mitochondrial quality control process relies on the identification of damaged or dysfunctional mitochondria for clearance [128]. This process, termed mitophagy, is a highly selective form of autophagy that begins with the recognition of depolarized and unhealthy mitochondria. This targeting step is regulated by PTEN-induced kinase 1 (PINK1), a protein kinase that accumulates and stabilizes on the OMM upon loss of mitochondrial membrane potential; this translocation leads to the phosphorylation of both ubiquitin and Parkin, an E3 ubiquitin ligase, at Ser(65), and Parkin recruitment to mitochondria [129,130]. Activated Parkin then initiates the ubiquitination of several substrates in the OMM, leading to the recruitment of autophagy adapter proteins such as p62, Optineurin (OPTN), and NDP52, which then initiates mitophagy. The clearance process proceeds with the formation of an autophagic membrane around the organelle (mitophagosome), which is later fused with a lysosome for degradation (mitophagolysosome). In this structure, lysosomal hydrolases digest mitochondria into small components that can then be recycled [131,132]. Besides PINK1–Parkin–ubiquitin-mediated mitophagy, the clearance of damaged mitochondria can also proceed with the involvement of receptor proteins. In mammalian cells, these receptors (e.g., NIP3-like protein X (NIX; also known as BNIP3L), FUN14 domain containing 1 (FUNDC1)) are transmembrane proteins attached to the OMM and must contain an LC3 motif, which allows the localization of the autophagosome with mitochondria [128]. Regardless of the mitophagy pathway, compelling studies have shown that this process plays a crucial role in regulating the machinery of the cell cycle, disposing of mitochondrial waste during mitosis, maintaining bioenergetic homeostasis, and preventing the accumulation of dysfunctional mitochondria that can lead to cellular degeneration. As such, mitophagy failure has been intrinsically related to a multitude of pathologies, being identified as a hallmark of age-related neurodegenerative disorders, including AD [132]. Indeed, different models of disease have consistently described the accumulation of damaged mitochondria as a fundamental event in AD pathogenesis [133,134]. In detail, immunohistochemical experiments allowed the observation of a significant reduction in the colocalization staining of TOMM20 (OMM protein) and LAMP2 (lysosomal protein), whilst electron microscopy images showed fewer mitophagy-like events in the postmortem hippocampal regions of AD patients relative to age-matched healthy controls, indicating mitophagy failure and the accumulation of compromised mitochondria in the hippocampi of AD patients [134]. An impairment in mitophagy was also observed in fibroblasts from sporadic AD individuals, as illustrated by a diminished number of autophagic vesicles (LC3) and inefficient Parkin translocation to the mitochondria, causing the accumulation of activated PINK1 [135]. Parallel in vivo and in vitro studies have contributed to better understanding the link between defective mitophagy and AD neuropathological hallmarks [135,136]. For instance, Cummins and colleagues found that tau protein expression directly inhibited mitophagy by aberrantly interacting with Parkin in cytosol and impeding its translocation to damaged mitochondria [136]. Notably, their experiments, which were performed in two different models, a neuroblastoma cell line and *Caenorhabditis elegans* neurons, showed that these effects occurred with both wild-type and frontotemporal dementia (P301L) mutant tau and did not require changes in mitochondrial membrane potential or the cytoskeleton [136]. Concurrently, others have described mitophagy deficits in HEK293 cells and primary hippocampal neurons overexpressing human tau, alongside reduced levels of PINK1/Parkin in the mitochondrial fraction [137]. However, differently from the previous study, these observations were reportedly linked to the allocation of the tau protein in the OMM, which caused increased mitochondrial membrane potential and blocked PINK1/Parkin mitochondrial location [137], highlighting the different possible mechanisms of tau-mediated mitochondrial toxicity. Besides the tau protein, two comparable studies highlighted a connection between the hippocampal accumulation of mutant APP and Aβ and reduced mitophagy in an animal model of AD and in an immortalized hippocampal transgenic cell line overexpressing mAPP [138,139]. More recently, Vaillant-Beuchot and co-workers uncovered that, independently of Aβ, APP-derived C-terminal fragment (APP-CTF) accumulation in mitochondria-enriched fractions can elicit basal mitophagy failure, apparently due to enhanced autophagy induction and the inefficient colocalization/targeting of mitochondria with/to lysosomes [140]. It was demonstrated that APP-CTF accumulation in the mitochondria of various AD study models caused the increased recruitment of PINK1/Parkin to mitochondria, the greater conversion of LC3, the accumulation of LC3-II, the non-degradation of the p62 substrate, enhanced levels of membrane and matrix mitochondrial proteins, and the deficient fusion of mitochondria with lysosomes [140]. Altogether, these findings point out the complex factors that can underlie mitophagy failure in AD pathology and pave the way for future interventions targeting this key process. 

## 3. Mitochondria-Based Therapies for Alzheimer’s Disease

**Figure 2:**
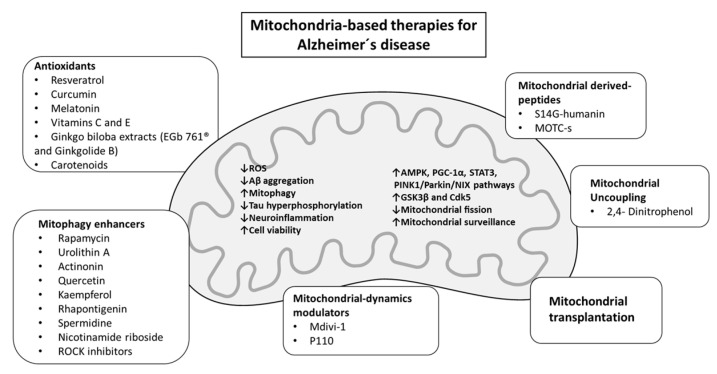
Simplistic representation of current strategies aimed at targeting Alzheimer´s disease faulty mitochondria. Regarding the Alzheimer´s disease (AD) pathophysiology, mitochondria decline arises as a common denominator in the majority of dedicated studies. In view of this evidence, preserving and/or restoring mitochondria’s health and function can represent the primary means to achieve advances to tackle AD. Thus far, a wealth of research has been dedicated to developing mitochondria-targeted therapeutics that could strengthen/restore the multifaceted functions of mitochondria to circumvent AD’s cascade of events. Herein, we show mitochondrial-directed antioxidants, mitochondrial-derived peptides, mitochondrial dynamics and mitophagy-targeting compounds, mitochondrial uncoupling, and mitochondrial transplantation as disease-modifying strategies in AD pathophysiology. Aβ—abeta peptide; AMPK—AMP-activated protein kinase; GSK3β—glycogen synthase kinase 3beta; PGC-1α—peroxisome proliferator activated receptor-gamma (PPARγ) coactivator-1alpha; PINK1—PTEN-induced kinase 1; ROCK—Rho-associated protein kinase; ROS—reactive oxygen species; STAT3—signal transducer and activator of transcription 3. ↑ Increase; ↓ Decrease.

Due to the high complexity and multifactorial nature of the disease, AD investigation comprises a vast portfolio of attempted approaches that in general have been unsuccessful. With the goal of challenging the conventional scenario of targeting Aβ, research is now starting to focus on strategies directed at improving/restoring the multifaceted functions of mitochondria (Figure 2 and Table 1).

### 3.1. Mitochondrial Antioxidant Interventions

As aforementioned, an important hallmark of the AD pathology and one of the earliest changes in AD brains is the occurrence of a chronic imbalance between antioxidant defenses and the production and accumulation of ROS. Thus, it has been theorized that counterbalancing the levels of oxidative stress via antioxidant supplementation could be beneficial in the prevention or delay of the cascade of AD-induced neurodegeneration [141]. One well-known antioxidant is resveratrol, a polyphenol non-flavonoid present in several edible plants, such as grapes, peanuts, and berries, found to exert significant effects on mitochondria and to have immense therapeutic prospects, including in AD, being already evaluated in controlled clinical trials (NCT01504854) [142]. From a mechanistic perspective, earlier studies show that resveratrol is able to protect against Aβ-induced neurotoxicity through inhibiting Aβ aggregation [143], stimulating mitophagy [144], activating important metabolic and signaling pathways (e.g., AMPK, SIRT1, and PKC) [145,146,147], stimulating cellular antioxidant defenses [148], and reducing the inflammatory responses of APP/PS1 mice associated with Aβ-induced microglial activation [149]. However, despite the beneficial effects, the data obtained in clinical trials were not consistent and highlighted some limitations, mainly related to the low systemic bioavailability of the antioxidant [150], which can hamper its distribution to brain mitochondria. To address these issues, pre-clinical research is starting to investigate methods to improve resveratrol’s tissue targeting, bioavailability, and efficacy [147,151,152]. For instance, in a recent study, Han and colleagues conducted experiments using a nanostructured lipid particle system (NPs@RBCm) able to specifically deliver resveratrol to neuronal mitochondria. Their data showed that the resveratrol-loaded novel biomimetic nanosystem therapy improved the cognitive ability of APP/PS1 mice, decreased Aβ levels, mitigated brain inflammation, and improved mitochondrial oxidative stress in the hippocampi of AD mice [153]. Nevertheless, the literature highlights that resveratrol’s effects on mitochondria are highly dependent on the dose and redox status of the experimental model [154]. In detail, Gueguen and colleagues demonstrated that resveratrol directly binds to complex I of the respiratory chain, increasing its activity; when administered to aged mice, this event promoted an imbalance in the oxidative status towards increased oxidative stress [154]. In further agreement, others reinforce the idea that resveratrol-mediated mitochondrial effects strongly depend on the concentration administered [155].

Another natural product with antioxidant potential is curcumin, a phenolic compound extracted from the root of *Curcuma longa* [156]. Curcumin is described to have pleiotropic pharmacological effects and has been used in the treatment of several different pathologies (e.g., cardiac diseases, rheumatism, asthma, hepatic disorders, among others) [157]. In the context of AD, several lines of evidence have shown that curcumin has anti-amyloid properties, influencing Aβ aggregation [158] and cellular uptake [159], downregulating Aβ production [160], and reducing plaque deposition [161]. Others have also demonstrated that curcumin can interfere with the tau pathology, avoiding tau protein hyperphosphorylation and tangle formation [162], attenuating inflammatory processes [163], and improving mitochondrial function [164,165]. However, similarly to resveratrol, curcumin presents low bioavailability, which complicates its translation to clinical trials [166]. In fact, the first clinical studies evaluating the effects of curcumin in patients with AD did not show significant benefits with curcumin administration [167,168,169]. More recently, new curcumin formulations (Longvida^®^ and Theracurmin) allowed for an improvement in the antioxidants´ bioavailability and achieved beneficial effects on attention and working memory tasks in a healthy older population [170] and in non-dementia adults that also presented decreases in Aβ and tau accumulation in brain regions modulating mood and memory [171]. In the same context, Gao and co-workers, using a neuronal mitochondria-targeted biomimetic engineered delivery nanosystem able to directly target curcumin to brain mitochondria, demonstrated positive outcomes upon intravenous administration in an AD mice model [172].

Concurrently with these observations, other considerable pre-clinical and clinical studies have demonstrated the potential of Ginkgo biloba extract EGb761^®^ to treat age-related cognitive disorders, especially AD [173]. EGb 761^®^, an extract made from the dry leaves of Ginkgo biloba, was found to have strong free radical scavenging and mitochondrial-protective properties [174], mechanisms that seem to underlie the described protective effects, such as improved neuronal function [175] and neuroinflammation [176], a shift in APP metabolism towards non-amyloidogenic pathways [177], Aβ toxicity, and cognitive performance [178]. In this context, presently, the antioxidant and anti-inflammatory effects of EGb 761^®^ are being evaluated in a Phase IV randomized, open-label clinical trial in MCI subjects to identify its possible clinical correlation with cognitive outcomes (NCT05594355) [179]. Complementing the existing data on to the neuroprotective effects of EGb 761^®^, recent studies have also investigated the effects of one of its main active components, Ginkgolide B, in the AD context. For example, evidence shows that Ginkgolide B is effective in reducing oxidative stress and protecting against Aβ toxicity [180], inactivating inflammasome formation [181], and modulating gut dysbiosis [182], allowing for improved cognitive function in animal models of AD [182,183]. Clinically, Ginkgolide B’s potential protective effects remain to be determined. 

Alongside the potential of the abovementioned natural products, the latest findings suggest that melatonin can also have therapeutic effectiveness as a mitochondria-targeted antioxidant in AD [184]. Melatonin is widely known for its sleep–wake cycle regulatory properties. However, data confirm that melatonin exists inside mitochondria and modulates their function and acts as a free radical scavenger and antioxidant defense stimulator and as an anti-inflammatory agent at an organ and system level [185]. Earlier studies demonstrated that the melatonin concentration was decreased in the cerebrospinal fluid of AD patients, which seemed to parallel the pathophysiological progression of the disease [186,187]. Since then, considerable pre-clinical studies have supported the potentiality of melatonin as a treatment strategy to inhibit AD pathogenesis [188,189,190,191]. For instance, it was observed that melatonin supplementation in 5xFAD mice restored the mitophagy process, ameliorated mitochondrial energy metabolism, and inhibited Aβ formation, resulting in significant improvements in the cognitive function of transgenic mice [192]. Translating such outcomes to the clinic, the first therapeutic trials suggested that melatonin could be effective in ameliorating sleep efficacy and total sleep time in patients with dementia [193] and in improving mini-mental state examination (MMSE) scores in mild AD patients [194]. In a more recent clinical study, melatonin intake improved the sleep onset period and sleep cycle in early-to-moderate AD patients [195]. In the same context, the use of add-on prolonged-release melatonin showed positive effects on sleep maintenance in mild-to-moderate AD patients, especially in those with comorbid insomnia [196]. 

Beyond these novel mitochondrial-directed antioxidative supplements, considerable pre-clinical evidence exists demonstrating the beneficial effects of conventional antioxidants like vitamins C and E [197] and carotenoids (for a review, see [198]) on AD-related pathology. However, whist vitamins C and E’s clinical effectiveness remains inconclusive and more clinical studies must be conducted to demonstrate their efficacy in an AD context [199], carotenoid intake has been associated with a lower risk of MCI in the Chinese elderly population [200], with a higher cognitive score in the general population (NCT00272428) [201] and with a lower risk of AD neuropathology in a community-based cohort of older adults [202]. More recently, the administration of a combined micronutrient dietary supplement containing carotenoids, omega-3 fatty acids, and vitamin E achieved positive outcomes regarding the cognitive performance and mood of mild–moderate AD individuals [203], emphasizing the relevance of antioxidant therapy to AD. 

### 3.2. Mitochondrial-Derived Peptides as Therapeutic Agents

The human mitochondrial DNA (mtDNA) has numerous short open reading frames (sORFs) that produce potential microproteins known as mitochondria-derived peptides (MDPs) [204]. The first MDP to be discovered was humanin. This 24-amino-acid peptide was first cloned from the resilient brain tissue of an AD patient by the group of Hashimoto et al., who identified it as an antagonizing factor against AD-associated neurotoxicity [205]. Later, Kelvin Yen and colleagues reported that humanin levels were decreased in the cerebrospinal fluid of AD patients, in comparison with controls, and a specific genetic variation in humanin sORF, m.2706A>G, was associated with more pronounced cognitive aging in African Americans [206]. Since then, multiple studies have been performed to assess the feasibility of humanin and its derivatives in the AD context. Using in vivo models of the disease, earlier evidence showed that the administration of S14G-HN, a more potent humanin derivative, ameliorated the cognitive performance of Aβ-injected [207] and 3xTg-AD [208] mice and reduced Aβ accumulation and neuroinflammation in the brains of middle-aged APP/PS1 mice [209]. Currently, it is known that the neuroprotective effects of humanin comprise its interaction with different cell surface receptors like the ciliary neurotrophic factor receptor (CNTFR) and the seven-transmembrane G-protein-coupled receptor formyl-peptide receptor-like-1 (FPRL1) [210]; the activation and modulation of the tyrosine kinase, JNK, AKT, ERK1/2, and STAT3 pathways as downstream effects [211]; and the prevention of mitochondria dysfunction and mitochondria-dependent apoptosis [212,213]. Complementing this evidence, it was also found that humanin can directly interact with Aβ, reducing its aggregation and oligomerization [214], and it is able to decrease the inactivation of phosphatase 2A that modulates tau pathology [206]. More recently, Han and colleagues showed that S14G-humanin-mediated protection in APP/PS1 transgenic mice entailed the regulation of autophagy, leading to the decreased accumulation of Aβ in the hippocampus and improved spatial learning and memory [215]. Beyond monotherapy, S14G-humanin has also been tested in a multifunctional hybrid peptide, HNSS, composed of S14G-humanin and the antioxidant peptide SS31, in 3xTg-AD mice [216]. It was observed that HNSS targeted mitochondria in the mouse brain, effectively rescuing mitochondria dysfunction via the PGC-1α and STAT3 pathways; neutralized AD-neurotoxic proteins, Aβ deposition, and tau hyperphosphorylation; and ameliorated memory defects and cholinergic neuronal damage in the 3xTg-AD mice [216]. Besides S14G-humanin, Colivelin has been identified as another potent humanin derivate able to improve the AD pathological damage and associated impairments in learning and memory that occurred in APP/PS1 mice [217] and to prevent Aβ_25–35_-induced deficits in spatial memory and synaptic plasticity in rats [218].

Alongside humanin, mitochondrial-derived peptides include MOTS-c (mitochondrial open reading frame of the 12S rRNA type-c) and six small humanin-like peptides (SHLP1-6) that differ in their expression patterns and ability to regulate cell viability and mitochondrial function [219]. Among these, SHLP2 was found to decline with age and, like humanin [206], was able to protect primary mouse cortical neurons from toxicity caused by Aβ_1–42_ [219]. Regarding MOTS-c, published evidence demonstrates that, under normal conditions, this 16-amino-acid peptide is found localized in mitochondria. However, in response to stress or exercise, it is rapidly translocated to the nucleus in a 5′-adenosine monophosphate-activated protein kinase (AMPK)/PGC1-α-dependent manner, where it promotes the expression of antioxidant and modifiable adaptive genes, improving redox homeostasis [220,221,222]. To further investigate the effect of MOTS-s on memory processes and the central inflammatory response under Aβ_1–42_ or LPS insults, an earlier study evaluated these outcomes using different routes of MOTS-c administration [223]. Their data showed that when administrated either by intrahippocampal CA1 microinjection or intracerebroventricular injection (icv), MOTS-s promoted the formation and consolidation of object and location recognition memory and attenuated Aβ_1–42_ or LPS-induced memory disability and the LPS-induced activation of microglia and astrocytes. In agreement with previous data, these effects were abolished when an inhibitor of the AMPK signaling pathway was administered prior to treatment. Importantly, the authors observed that MOTS-c did not cross the blood–brain barrier (BBB) and so, when administered peripherally, it was not able to exert neuroprotection. To circumvent such a drawback, a cell-penetrating MOTS analogue was designed and synthesized, demonstrating efficacy when intranasally injected in Aβ_1–42_ or LPS-icv-treated mice [223]. Notably, despite the favorable outcomes obtained with pre-clinical models, mitochondrial-derived peptides’ safety and efficacy have not been clinically tested in the context of AD. Presently, CB4211, a MOTS-c peptide analogue, is being evaluated in a Phase 1a double-blind, placebo-controlled clinical trial in obese subjects with non-alcoholic fatty liver disease (NAFLD) (NCT03998514).

### 3.3. Mitochondrial Dynamics and Mitophagy-Targeting Therapy in AD

As stated above, compelling research demonstrates that disrupted mitochondrial dynamics, biogenesis, and mitophagy are important and interconnected factors associated with the loss of neuronal cells’ homeostasis in AD. Such evidence opens up a new perspective on the beneficial effects that could stem from interventions that maintain and/or enhance mitophagy and mitochondrial structural and functional integrity in AD pathophysiology [224,225].

#### 3.3.1. Mitochondria Dynamics Modulators

Under physiological conditions, the mitochondrial shape can quickly range from small round structures to elongated tubular networks due to the tightly coordinated action of mitochondrial fusion–fission proteins that rapidly react to challenges [84]. However, under pathological contexts, as is the case in AD, the mitochondrial dynamics’ equilibrium is disrupted, presenting a bias towards increased mitochondrial fragmentation due to increased levels of DRP1 [226]. In response to this, studies have been exploring the potentiality of targeting and inhibiting mitochondrial fission [227]. The most well-known compound is Mdivi-1. Discovered in the late 2000s as a potent inhibitor of DRP1 [228,229], Mdivi-1 treatment has been found to improve learning and memory processes, to rescue mitochondrial morphology, and to inhibit lipid peroxidation, BACE1 expression, and Aβ deposition in the brains of APP/PS1 mice [230]. Concurrently, others have reported the positive effects of Mdivi-1 in reverting Aβ-induced excessive mitochondrial fragmentation and synaptic toxicity in a mouse neuroblastoma (N2a) cell line [231].

Using the same rationale, P110 was developed as a new peptide able to inhibit mitochondrial fragmentation [232]. However, differently from Mdivi-1, P110 acts by disrupting the DRP1/Fis1 interaction, without affecting the integrity of the mitochondrial network in the basal state and the interaction of DRP1 with other adaptors [232]. In pre-clinical studies, P110 administration promoted neuroprotective outcomes such as the improvement of mitochondrial health, a reduction in Aβ levels, and the restoration of cognitive function in AD animal models [86]. Moreover, it was recently reported that P110, via the inhibition of DRP1/Fis1 interaction, was able to reduce the release of damaged mitochondria from neurotoxin-activated microglia and the consequent activation of the inflammatory response, protecting neuronal cells [233]. Nevertheless, despite the beneficial effects reported so far, there are no clinical data to document the efficacy of mitochondria dynamics modulators in AD.

#### 3.3.2. Mitophagy Enhancers

One of the most well-known mitophagy-stimulating agents is rapamycin, an inhibitor of the mTOR pathway [234]. According to a study carried out on a mutant APP transgenic mouse model, the rapamycin-mediated inhibition of mTOR can ameliorate Aβ pathology and cognitive dysfunction and either slow down or halt the progression of AD [235]. Concurrently, others showed that rapamycin treatment could reverse AD-like pathology, mitochondrial abnormalities, and cognitive deficits via decreasing mTOR signaling in the hippocampus in a diabetic mice model [236]. Similarly, experimental evidence showed that rapamycin administration in young 3xTg-AD mice increased autophagy and decreased soluble Aβ and tau pathology [237]. Meanwhile, the same group observed that although rapamycin had beneficial effects when administered to young 3xTg-AD mice, in contrast, its administration in older mice had no effects on AD-like pathology and cognitive deficits [238]. In this regard, a very recent study by Shi and colleagues [239] demonstrated that rapamycin-induced mTOR pathway inhibition in 5XFAD mice significantly compromised microglia Aβ plaque clearance, corroborating the notion that rapamycin use in AD patients should be carefully analyzed [240]. At present, rapamycin’s safety and tolerability are being evaluated in a clinical trial in older adults with amnestic MCI (aMCI) and early-stage AD (NCT04629495).

Alongside rapamycin, research has been focused on evaluating the therapeutic potential of several pharmacological agents and natural supplements that could target mitophagy and improve mitochondrial function in the AD context [241]. In this regard, urolithin A (UA), a by-product of ellagitannin polyphenols occurring in pomegranate and walnuts, has demonstrated a significant ability to enhance cellular health by promoting mitochondrial function normalization and mitophagy and reducing detrimental inflammation in several pre-clinical models of aging and disease [242]. Regarding the clinical setting, the first clinical trial with UA showed that the regular oral consumption of UA in sedentary elderly individuals had a favorable safety profile (primary outcome) and led to an enhancement in mitochondrial and cellular molecular signs of fitness [243]. Corroborating data were obtained in a recent proof-of-concept investigation in which the benefits of the longer consumption of UA were assessed in middle-aged adults in terms of several physiological and biomarker endpoints [244]. Results obtained showed that UA promoted higher mitochondrial efficiency and mitophagy and reduced inflammation in the skeletal muscles of participants [244]. In cellular and animal models of AD, studies confirmed that UA lessened tau hyperphosphorylation in human neuronal cells and reversed memory impairments in transgenic nematodes and mice models [134,245]. Similar effects were observed with actinonin, an antibiotic able to trigger mitophagy in a PINK1/Parkin/NIX-dependent manner, and normalize mitochondria function and morphology in AD animal models [134]. Further, others report that UA’s beneficial effects can be enhanced when administered in combination with a green tea extract (epigallocatechin gallate, EGCG) [246]. Using the humanized Aβ knockin mice (hAbKI) as an experimental model, Kshirsagar and colleagues reported that the combined administration of UA+EGCG targeted multiple mechanisms of disease, such as inflammation, oxidative stress, synaptic loss, Aβ accumulation, cognitive deficits, and mitochondria homeostasis, thus suggesting the benefits of a combined approach instead of a monotherapy for AD [246]. In line with this, the combination of baicalein with memantine, one of the current U.S. FDA-approved drugs for AD, with an ability to enhance autophagic flux and the clearance of damaged mitochondria [247], showed enhanced therapeutic potential in Aβ-induced AD in Wistar rats [248] due to its ability to reach multi-therapeutic targets, demonstrating promising translational outcomes [248]. Previous studies show that flavonoids can have neuroprotective properties [249]. For instance, quercetin, a natural flavonoid that can be found in fruits, vegetables, berries, grapes, wine, various seeds, and nuts, has the ability to cross the BBB and modulate the expression and activity of PINK1/Parkin-mediated mitophagy and stimulate cellular defenses against oxidative stress, promoting positive effects in neurodegenerative contexts [250,251]. Compelling data from in vitro and in vivo models confirm the therapeutic efficacy of quercetin in modulating AD neuropathology in various experimental models of the disease [252,253,254]. More recently, Xie and co-workers [255], using combined advanced artificial intelligence with classical wet laboratory approaches, identified kaempferol, a natural flavonol, and rhapontigenin, a natural product and analog of resveratrol, as two potent mitophagy inducers that could be potential drug candidates for AD treatment. In their work, both compounds exhibited positive outcomes in one Aβ nematode model, two Tau nematode models, and the 3xTg-AD mouse model. 

Spermidine is another versatile and multipurpose natural compound that can provide benefits for aging and age-related diseases like AD [256]. As shown in a recent pilot trial, spermidine supplementation for three months had a positive impact on memory performance in older adults at risk for the development of AD (NCT02755246) [257]. Recent evidence suggests that the maintenance of mitochondrial and autophagic function is the main mechanism of spermidine-mediated neuroprotection [258,259]. As noticed, spermidine was able to rescue the mitochondrial function of SH-SY5Y cells expressing a mutant form of the human tau protein (P301L tau mutation) [260]. As an autophagy inducer, spermidine modulates the mTOR and AMPK pathways [261] and activates Ataxia Telangiectasia mutated (ATM)-dependent PINK1/Parkin signaling [262].

Extensive studies using pre-clinical models of AD have demonstrated that increasing nicotinamide adenine dinucleotide (NAD^+^) levels through supplementation with NAD^+^ precursors (e.g., nicotinamide and its derivatives nicotinamide riboside (NR) and nicotinamide mononucleotide (NMN)) stimulates mitophagy; mitigates Aβ, tau, and impaired energy metabolism; and modulates the inflammatory response, improving cognitive function [263,264,265,266]. Based on this, different clinical trials are currently being performed to evaluate the impact of NAD^+^ supplementation on brain function, cognition, oxidative stress, bioenergetics metabolism, and CSF pTau levels in MCI and AD patients (NCT03482167; NCT04430517; NCT04078178; NCT03061474). Following this rationale, the efficacy of combined metabolic activators (CMA) in AD patients is being evaluated in a Phase II clinical study (NCT04044131) [267]. CMA consists of the combination of L-serine, N-acetyl cysteine (NAC), nicotinamide riboside (NR), and L-carnitine tartrate (LCAT, the salt form of L-carnitine), and its oral administration to AD patients showed positive effects on cognitive function and markers of metabolic abnormalities, even in patients with severe AD [267]. 

Apart from the use of bioavailable neuronal mitophagy inducers, recent studies have identified a serine-threonine kinase (Rho-associated protein kinase 2; ROCK2) as a negative regulator of Parkin-dependent mitophagy [268]. ROCK2, which was firstly identified as a main regulator of the actin cytoskeleton and dendritic branches [269], is expressed in the cerebral cortex, hippocampal neurons, and cerebellar Purkinje cells, among other regions of the brain [270], in an age-dependent way. It was observed that brain sections from older human subjects possessed a higher number of ROCK2-positive cells as compared to younger controls [271]. In this regard, the application of ROCK inhibitors was found to improve synaptic processes and ameliorate rodents’ memory and cognitive behavior [272,273]. To investigate the applicability of ROCK inhibitors in AD, Hamano and colleagues [274] treated M1C cells (a human neuroblastoma cell line) overexpressing wild-type tau protein (4R0N) and a mouse model of tauopathy with three different ROCK inhibitors, H1152, Y-27632, and fasudil (HA-1077). The authors observed that ROCK inhibitors downregulated the activity of tau protein’s main kinases (GSK3β and Cdk5), reduced total tau levels, and upregulated autophagy [274]. In close agreement, ROCK knockdown in SH-SY5Y cells or its pharmacological inhibition in primary culture neurons contributed to a significant reduction in both tau mRNA and protein levels and to the decreased activation of mTOR, leading to the regulation of autophagy [275], thus supporting the clinical use of ROCK inhibitors as rational therapeutic approaches for AD and other tauopathies (NCT04734379). 

Overall, these findings support the notion that strategies targeted at normalizing the balance between healthy and damaged mitochondria, by promoting the clearance of damaged and fragmented mitochondria, represent suitable candidates to provide neuronal protection in neurodegenerative diseases like AD.

### 3.4. Mitochondrial Uncoupling in AD

Alongside antioxidants, mitochondria are equipped with inner mitochondrial proteins able to counteract the production of free radicals [276]. These remarkable proteins, called mitochondrial uncoupling proteins (UCPs), exist in five isoforms depending on the organ in which they are expressed and serve the main purpose of transferring the protons produced during electron flow within the ETC against the concentration gradient. This, in turn, uncouples the process of electron transport from the synthesis of ATP through oxidative phosphorylation, allowing the regulation of the production of O_2_^•−^ [277,278]. As reviewed elsewhere, whilst complete uncoupling would dissipate the cellular ATP pools, a small reduction in the mitochondrial membrane potential induced by mild uncoupling does not compromise the overall net ATP and has a significant effect in ameliorating ROS production [279]. Among the different isoforms, UCP2, UCP4, and UCP5 are the main UCPs detected in the brain [280]. Several studies suggest that brain UCPs play a key role against oxidative-stress-related brain pathologies, as is the case of AD, mainly through the modulation of ROS generation [281,282,283]. In fact, it has been shown that UCP2 protects hippocampal neurons against Aβ_1–40_-induced oxidative stress and toxicity [284]. Consistently, it was found that AD brain postmortem samples had lower expression levels of UCP2 and UCP4 when compared with samples from non-AD brains [285]. Furthermore, it has been reported that UCP2’s effects are not restricted to neurons but can also extend to other brain cells, such as microglia and astrocytes. For example, in LPS-stimulated microglia, UCP2 silencing caused an increase in M1 gene expression and an enhanced inflammatory response [286]. In line with this, Thangavel et al. demonstrated that AD brains presented increased expression of the proinflammatory protein glia maturation factor, which seemed to be related to the downregulation of both UCP2 and UCP4 expression and an increased proinflammatory environment in the parahippocampal gyrus in AD brains as compared to non-AD brains [285]. In this context, Rosenberg et al. demonstrated that the targeted overexpression of UCP4 in astrocytes’ mitochondria prevented hippocampal atrophy, neurons’ dendritic shrinkage, metabolic alterations, and spatial memory deterioration in a mouse model of AD [287]. These results suggest that modulating UCPs’ expression/activity in brain cells allows us to re-establish mitochondrial homeostasis and AD pathological outcomes. This may indicate that targeting the uncoupling mechanism is an approach worth investigating in AD treatment [288]. One well-known example of a pharmacological uncoupler able to mimic UCP-mediated brain effects is 2, 4-dinitrophenol (DNP). Described as a fast weight reduction agent, DNP is a chemical uncoupler that allows protons’ re-entrance to the mitochondrial matrix, thus interfering with ATP synthesis and mitochondrial membrane potential [289]. Compelling data suggest that DNP-mediated effects are dose-dependent [289]. This means that when used at high concentrations, DNP can evoke serious side effects, such as agranulocytosis, hyperthermia, skin reactions, and cataracts; in contrast, when used at low concentrations, it induces mild mitochondrial uncoupling that is beneficial in terms of reducing oxidative neuronal damage in different pathological conditions, including AD [290,291]. The first evidence came from the group of De Felice, who observed that DNP, at low concentrations, protected neurons against Aβ toxicity [292] and promoted neuritogenesis and neuronal differentiation in cortical and hippocampal neuronal cultures [293]. In this context, later studies showed that DNP was able to decrease the intracellular accumulation of APP in an immortalized cell line derived from the cerebral cortex of an animal model of Down’s syndrome, which presented a pathophysiology closely related to AD [294]. More recently, others observed that the peripheral administration of DNP resulted in the complex adaptive remodeling of the molecular pathways that regulate neuronal stress responses and synaptic plasticity [295]. In line with this, it was shown that DNP administration in APP/PS1 mice increased hippocampus-dependent spatial learning and memory [289]. Collectively, these data suggest the importance of examining the clinical potential of targeting uncoupling proteins and/or uncoupling agents as a disease-modifying strategy in AD pathophysiology. 

**Table 1:** Overview of mitochondria-based therapies for Alzheimer’s disease.

Mitochondria-Based Therapeutics		Targets and Mechanism of Action	Benefits and Limitations	References
Antioxidants	Resveratrol	- Activates AMPK, SIRT1, and PKC signaling pathways - Binds complex I of the respiratory chain and increases its activity	**Benefits (pre-clinical):** - Stimulates cellular antioxidant defenses - Reduces the inflammatory response - Stimulates mitophagy - Inhibits Aβ aggregation **Limitations:** - Low systemic bioavailability - Mitochondrial effect strongly depends on the concentration administered and redox status of the experimental model **Benefits (clinical):** - Safe, well tolerated - Modulates neuroinflammation - Attenuates declines in mini-mental status examination (MMSE) scores - Alters AD biomarkers (NCT01504854)	[142,143,144,145,146,147,148,149,150,154,155]
	Curcumin	- Anti-Aβ and anti-tau properties	**Benefits (pre-clinical):** - Attenuates inflammatory processes - Improves mitochondrial function **Limitations:** - Low systemic bioavailability **Benefits (clinical):** Positive outcomes on attention and working memory tasks	[158,159,160,161,162,163,164,165,166,170,171]
	Melatonin	- Free radical scavenger - Inhibition of Aβ formation	**Benefits:** - Stimulates antioxidant defenses - Attenuates neuroinflammation - Restores mitophagy process - Ameliorates mitochondrial energy metabolism - Improves cognitive function **Benefits (clinical):** - Ameliorates sleep efficacy and total sleep time - Improves mini-mental state examination (MMSE) score	[185,190,191,192,193,194,195,196]
	Vitamins C and E	- Free radical scavengers - Neutralization of lipid peroxidation products - Mitochondrial protectants	**Benefits:** - Antioxidant effect **Limitations:** - Inconclusive clinical studies	[197,199]
**a**	Carotenoids	- Antioxidative properties - Anti-inflammatory	**Benefits (clinical):** - Lower risk of MCI - Better cognitive scores - Amelioration of AD neuropathology	[200,201,202]
	Ginkgo biloba extracts (EGb761^®^ and Ginkgolide B)	- Free radical scavengers - Mitochondria protectants - Anti-inflammatory - Anti-amyloidogenic effect	**Benefits (pre-clinical):** - Stimulate antioxidant defenses - Attenuate neuroinflammation - Ameliorate mitochondrial function - Improve cognitive function **Benefits (clinical):** - Improve neuropsychiatric symptoms - Improve cognitive function	[175,176,177,178,179,180,181,182,183]
Mitochondrial-derived peptides	S14G-humanin	- Interacts with different cell surface receptors - Activates and modulates tyrosine kinase, JNK, AKT, ERK1/2, and STAT3 pathways	**Benefits (pre-clinical):** - Reduces Aβ aggregation and oligomerization - Modulates tau pathology - Regulates autophagy - Improves cognitive performance	[206,211,214,215]
	Colivelin	- Humanin derivative	**Benefits (pre-clinical):** - Improves AD pathology - Recovers cognitive performance	[217,218]
	MOTC-s	- Benefits (pre-clinical): - Activates AMPK/PGC1-α pathway - Modulates redox response	**Benefits (pre-clinical):** - Attenuates Aβ_1–42_ or LPS-induced memory disability **Limitations:** - Does not cross the blood–brain barrier	[220,221,222,223]
Mitochondrial dynamics modulators	Mdivi-1	- Inhibits mitochondrial fission/DRP1	**Benefits (pre-clinical):** - Rescues mitochondrial morphology - Inhibits lipid peroxidation - Improves learning and memory - Inhibits Aβ deposition - Counteracts Aβ-induced excessive mitochondrial fragmentation	[230,231]
	P110	- Disrupts DRP1/Fis1 interaction	**Benefits (pre-clinical):** - Improves mitochondrial - health - Reduces Aβ levels - Restores cognitive function - Reduces inflammatory response	[85,233]
Mitophagy enhancers	Rapamycin	- Inhibits mTOR pathway	**Benefits (pre-clinical):** - Ameliorates Aβ pathology - Improves cognitive dysfunction - Rescues mitochondrial morphology - Decreases soluble Aβ and tau pathology **Limitations (pre-clinical):** - Effects depend on the age of the experimental model - Can compromise microglia Aβ plaque clearance **Benefits (clinical):** Under evaluation (NCT04629495)	[235,236,238,239]
	Urolithin A	- Normalizes mitophagy	**Benefits (pre-clinical):** - Ameliorates AD pathology - Improves cognitive dysfunction - Restores mitochondrial function - Reduces inflammation **Benefits (clinical):** - Favorable safety profile - Enhances mitochondrial and cellular molecular signs of fitness - Reduces inflammation markers	[134,242,243,244,245]
	Actinonin	- Activates PINK1/Parkin/NIX pathway	**Benefits (pre-clinical):** - Restores mitochondria function and morphology	[134]
	Memantine	- Modulates autophagy flux	**Benefits (pre-clinical):** - Favors the clearance of damaged mitochondria	[247]
	Quercetin	- Modulates the expression and activity of PINK1/Parkin-mediated mitophagy - Stimulates cellular defenses against oxidative	**Benefits (pre-clinical):** - Modulates AD neuropathology	[250,251,252,253,254]
	Kaempferol and rhapontigenin	- Mitophagy inducers	**Benefits (pre-clinical):** - Rescue AD pathology	[255]
	Spermidine	- Modulates mTOR and AMPK pathways - Activates ataxia telangiectasia mutated (ATM)-dependent PINK1/Parkin signaling	**Benefits (pre-clinical):** - Rescues mitochondrial function **Benefits (clinical):** - Improves memory performance (NCT02755246)	[257,260,261,262]
	Nicotinamide adenine dinucleotide (NAD^+^) percursors	- Increase nicotinamide adenine dinucleotide (NAD^+^) levels	**Benefits (pre-clinical):** - Reduce inflammation markers - Stimulate mitophagy - Mitigate Aβ, tau pathology - Rescue energy metabolism - Improve cognitive function **Benefits (clinical):** - Under evaluation (NCT03482167; NCT04430517; NCT04078178; NCT03061474)	[263,264,265,266]
	ROCK inhibitors	- Negative regulators of Parkin-dependent mitophagy	**Benefits (pre-clinical):** - Downregulate the activity of tau protein’s main kinases (GSK3β and Cdk5) - Reduce total tau levels - Upregulate autophagy - Regulation of autophagy **Benefits (clinical):** - Under evaluation (NCT04734379)	[274,275]
	Combined metabolic activators (CMA)	- Combination of L-serine, N-acetyl cysteine (NAC), nicotinamide riboside (NR), and L-carnitine tartrate (LCAT, the salt form of L-carnitine)	**Benefits (clinical):** - Positive effects on cognitive function and markers of metabolic abnormalities (NCT04044131)	[267]
Mitochondrial uncoupling	Mitochondrial uncoupling proteins(UCP2, UCP4)	- Promote mild mitochondrial uncoupling - Modulate ROS production and reduce oxidative damage	**Benefits (pre-clinical):** - Protect against Aβ_1–40_-induced oxidative stress and toxicity - Regulate inflammatory response - Re-establish mitochondria homeostasis - Prevent neuronal cells’ atrophy and loss	[279,281,282,283,284,286,287]
	2,4-Dinitrophenol	- Mimics UCP effects - Promotes mild mitochondrial uncoupling - Reduces oxidative damage - Induces adaptive responses	**Benefits (pre-clinical):** - Protects neurons against Aβ toxicity - Promotes neuritogenesis and neuronal differentiation - Decreases the intracellular accumulation of APP - Increases spatial learning and memory **Limitations (pre-clinical):** - Effects are dose-dependent	[280,281,282,283,284,285]

## 4. Mitochondrial Transplantation: Could the Transfer of Healthy Mitochondria Be the Solution?

As further reported herein, a great deal of effort has been dedicated to achieving a successful mitochondria-based therapy able to tackle AD. However, to date, only a few approaches have been able to yield satisfactory results, revealing limited translatability. Thus, alongside therapeutic interventions, an alternative perspective would be to look to mitochondria as therapeutics themselves [296]. Based on the knowledge that mitochondria are extremely dynamic organelles that can move between different cells (for a review, see [297,298]), the transfer of functional mitochondria to replace disabled mitochondria is starting to be considered a feasible option as a basis for cell therapy in a plethora of neural and non-neural disorders [299,300,301,302]. One of the earliest pieces of evidence of intercellular mitochondrial transfer came from an in vitro study in which the authors observed that the co-culture of mtDNA mutated and depleted cells (A549 ρ° cells) with mesenchymal stem cells allowed them to rescue the mitochondrial function of A549 ρ° cells [298]. Subsequent studies provided evidence for the potential of transferring healthy mitochondria to cells with mitochondrial defects [303,304,305,306,307]. Nowadays, this process, referred to as mitochondrial transplantation, is commonly achieved by isolating viable mitochondria from healthy tissue or cell lines and transferring them into the compromised recipient, e.g., an organ, in vivo model, or in vitro system [296]. Once inside the diseased recipient cells, exogenous organelles are believed to rescue the cells’ viability and, consequently, to avert disease onset and/or progression [308]. As a proof-of-concept trial, the first clinical application of mitotherapy was conducted in pediatric patients who suffered from myocardial ischemia–reperfusion injury [309]. In this study, a single epicardial injection of freshly isolated autologous mitochondria from skeletal muscle promoted patients’ myocardial recovery and improved ventricular function within several days of treatment. Importantly, as the authors noted, mitochondria autotransplantation did not cause any adverse heart-related side effects or markers of a systemic inflammatory response [309]. Since this pioneering work, other studies have been registered at ClinicalTrials.gov to evaluate the efficacy of this unique therapeutic, mainly in ischemia-related conditions (NCT03639506, NCT02851758, NCT05669144, and NCT04998357). 

Within the brain, previous studies demonstrate that mitochondria transfer can occur in a cell-to-cell manner between co-incubated astrocytes and neurons, as an endogenous strategy to safeguard neuronal survival after neurological damage [310,311]. More recently, others have observed that, alongside functional mitochondria, astrocytes also secrete the mitochondrial-genome-encoded small bioactive peptide humanin that is transported to microglia, reducing proinflammatory outcomes [312]. Notably, the efficacy of mitochondria transfer is intrinsically related to the age of the donor tissue/cell, as young mitochondria show better performance than older ones [313]. Corroborating these findings, mitotherapy with healthy young mitochondria obtained from the livers of young mice showed positive results in restoring the hippocampal mitochondrial bioenergetics of aged mice [314]. In a different setting, others demonstrated that the intravenous injection of hippocampus-derived isolated mitochondria in a status epilepticus (SE) disease model contributed to ameliorating hippocampal damage following SE and improved cognitive and mood dysfunction [315]. Their results showed that peripherally injected exogenous mitochondria could cross the BBB and revert SE-induced hippocampal injury by not only lessening SE-induced ROS production but also affecting the expression and metabolic pathways of important metabolites [315]. 

In the field of AD, mitochondrial transplantation as a therapeutic strategy is still in its infancy, but some recent pre-clinical research in experimental models of disease reports positive results. Using an in vitro model of disease established using okadaic acid (OA)-treated SH-SY5Y cells, Zhang and colleagues recently showed that a human-umbilical-cord-derived mesenchymal-stem-cell-conditioned medium (MSC-CM) exerted protective effects on AD through extracellular vesicles’ (EV) mitochondrial transfer [316]. The authors observed that treatment with MSC-CM significantly improved cell viability, apoptosis, and mitochondrial function in OA-treated SH-SY5Y cells via the donation of hucMSCs’ healthy mitochondria [316]. An additional study in an AD mice model achieved by the intracerebroventricular injection of the Aβ peptide showed that the intravenous injection of HeLa-cell-derived freshly isolated mitochondria contributed to ameliorating mice’s cognitive performance, hippocampal neuronal loss, and gliosis and reestablished mitochondrial functional outcomes [317]. More recently, a study conducted by Bobkova and collaborators verified that isolated mitochondria could be intranasally administered, leading to the recovery of spatial memory in olfactory bulbectomized (OBX) mice with AD-like degeneration [318]. Their data showed that functional isolated mitochondria from the brains of control mice, when intranasally injected, were detected in the hippocampus, neocortex, and olfactory bulbs in treated mice in a dose-dependent manner [318]. In a comparable manner, the same approach was evaluated in a mouse model of cisplatin-induced cognitive deficits. As the authors observed, nasally administered mitochondria isolated from human MSCs were able to reach brain meninges within 30 min of delivery, gaining access to the brain and reversing cisplatin-induced synaptic loss and hippocampal synaptosomal mitochondrial abnormalities [301], thus confirming the efficacy of mitochondria in crossing the BBB and restoring the homeostasis of brain cells. Overall, although some aspects of the efficacy, delivery, and safety concerns regarding mitochondria transplantation still need to be further explored [296], the remarkable outcomes reported in the literature strongly highlight the therapeutic prospects of mitotherapy in AD. 

## 5. Final Remarks

Despite decades of investigation, the understanding of the AD pathophysiology remains incomplete. In this scenario, mitochondria, or the decline in mitochondrial function, appears as a common denominator in the majority of dedicated studies. As the world approaches a new milestone in the number of diagnosed AD cases, and developing new medicines is a challenging and slow process, the consideration of mitochondria as a target holds great promise in AD therapy. It is important to note, though, that the development/refinement of the presently reviewed mitochondria-based therapeutics will require a multidisciplinary approach, including the identification of specific mitochondrial targets, a deeper understanding of how the tested substances affect the interactions between mitochondria and AD hallmarks (e.g., Aβ and tau) and consequently their toxicity, the optimization of drug delivery, and the development of new imaging techniques to monitor the effectiveness of the therapy in order achieve its full potential [319]. The early occurrence of mitochondrial impairment in AD pathophysiology, the late diagnosis of the disease, and the existence of other comorbidities (e.g., diabetes) also stand as important factors contributing to the complexity of the exploration of mitochondrial targeting approaches in AD. In this regard, besides the therapeutics/strategies mentioned in this manuscript, other pharmacological agents have been considered as feasible therapeutic options for AD. For instance, metformin, which is a commonly prescribed anti-diabetic medication, has been suggested to improve cognitive performance in AD individuals [320] and to rescue human neural stem cells from Aβ-mediated mitochondrial deficiency [321]. Additionally, some compounds, like tricaprilin, intended to regulate metabolism/bioenergetics, have also demonstrated efficacy in the management of mild to moderate AD [322]. Being currently tested in clinical trials (NCT00142805), tricaprilin seems to serve as an alternative fuel source for glucose, inducing ketosis and improving patients’ metabolism [322].

Thus, despite being a complex field, the positive outcomes of pre-clinical studies and of some clinical trials suggest that the development of mitochondrial-targeted therapeutics for AD is a promising research area that has the potential to lay the groundwork for new treatment options for this debilitating disease. Finally, considering the importance of mitochondria, it is imperative to understand that, regardless of the targeted disease mechanism, if the drug candidate compromises mitochondrial function, its possible clinical application could be at risk.
